# Leveraging sequences missing from the human genome to diagnose cancer

**DOI:** 10.1038/s43856-025-01067-3

**Published:** 2025-08-21

**Authors:** Ilias Georgakopoulos-Soares, Ofer Yizhar-Barnea, Ioannis Mouratidis, Candace S. Y. Chan, Michail Patsakis, Akshatha Nayak, Rachael Bradley, Mayank Mahajan, Jasmine Sims, Dianne Laboy Cintron, Ryder Easterlin, Julia S. Kim, Emmalyn Chen, Geovanni Pineda, Guillermo E. Parada, John S. Witte, Christopher A. Maher, Felix Feng, Ioannis Vathiotis, Nikolaos Syrigos, Emmanouil Panagiotou, Andriani Charpidou, Konstantinos Syrigos, Jocelyn Chapman, Mark Kvale, Martin Hemberg, Nadav Ahituv

**Affiliations:** 1 Department of Bioengineering and Therapeutic Sciences, University of California San Francisco, San Francisco, CA USA; 2 Institute for Human Genetics, University of California San Francisco, San Francisco, CA USA; 3 Institute for Personalized Medicine, Department of Molecular and Precision Medicine, The Pennsylvania State University College of Medicine, Hershey, PA USA; 4 Huck Institutes of the Life Sciences, Penn State University, University Park, PA USA; 5 The Gene Lay Institute of Immunology and Inflammation, Brigham and Women’s Hospital, Massachusetts General Hospital and Harvard Medical School, Boston, MA USA; 6 Department of Epidemiology and Biostatistics, University of California San Francisco, San Francisco, CA USA; 7 Division of Gynecologic Oncology, University of California San Francisco, San Francisco, CA USA; 8 Donnelly Centre for Cellular and Biomolecular Research, University of Toronto, Toronto, ON Canada; 9 Department of Epidemiology and Population Health, Stanford University, Stanford, CA USA; 10 Division of Oncology, Department of Internal Medicine, Washington University School of Medicine, St. Louis, MO USA; 11 Siteman Cancer Center, Washington University School of Medicine, St. Louis, MO USA; 12 Department of Biomedical Engineering, Washington University School of Medicine, St. Louis, MO USA; 13 Division of Hematology/Oncology, Department of Medicine, University of California San Francisco, San Francisco, CA USA; 14 Helen Diller Comprehensive Cancer Center, University of California San Francisco, San Francisco, CA USA; 15 Department of Radiation Oncology, University of California San Francisco, San Francisco, CA USA; 16 Department of Urology, University of California San Francisco, San Francisco, CA USA; 17 Third Department of Internal Medicine, Sotiria Hospital, National and Kapodistrian University of Athens, School of Medicine, Athens, Greece; 18 Department of Pathology, Yale School of Medicine, New Haven, CT USA; 19 Breast Oncology, Dana-Farber Brigham Cancer Center, Boston, MA USA; 20 Kaiser Permanente, Oakland, CA USA; 21 Wellcome Sanger Institute, Hinxton, Cambridge, UK; 22 Helen Diller Family Comprehensive Cancer Center, University of California San Francisco, San Francisco, CA USA

**Keywords:** Cancer genomics, Cancer epigenetics

## Abstract

**Background:**

Cancer diagnosis using cell-free DNA (cfDNA) has the potential to improve treatment and survival but has several technical limitations.

**Methods:**

In this study, we developed a prediction model based on neomers, DNA sequences 13–17 nucleotides in length that are predominantly absent from the genomes of healthy individuals and are created by tumor-associated mutations.

**Results:**

We show that neomer-based classifiers can accurately detect cancer, including early stages, and distinguish subtypes and features. Analysis of 2577 cancer genomes from 21 cancer types shows that neomers can distinguish tumor types with higher accuracy than state-of-the-art methods. Generation and analysis of 465 cfDNA whole-genome sequences demonstrates that neomers can precisely detect lung and ovarian cancer, including early stages, with an area under the curve ranging from 0.89 to 0.94. By testing various promoters or over 9000 candidate enhancer sequences with massively parallel reporter assays, we show that neomers can identify cancer-associated mutations that alter regulatory activity.

**Conclusions:**

Combined, our results identify a sensitive, specific, and simple cancer diagnostic tool that can also identify cancer-associated mutations in gene regulatory elements.

## Plain language summary

DNA fragments circulating in the blood are often present in people with cancer. Current tests using DNA fragments circulating in the blood have limitations in detecting cancer. We developed a cancer diagnostic approach that uses neomers, short DNA sequences that are mostly absent in healthy individuals but are present in tumors as a consequence of changes in the DNA (DNA mutations) in cancer cells. We show that this approach can accurately detect lung and ovarian cancers from DNA fragments extracted from blood and identify changes in the DNA associated with cancer. Our diagnostic tool could be used to improve cancer detection and identify how changes in the DNA affect development of cancer.

## Abstract (web summary)

Georgakopoulos-Soares, Yizhar-Barnea, Mouratidis et al. identify neomers, short (16 base pair) DNA sequences that are absent in the genomes of healthy individuals but appear in tumors due to mutations. They show that neomers can accurately detect cancer from cell-free DNA and that neomers found in gene regulatory elements can alter regulatory activity.

## Introduction

Cancer is the second leading cause of death worldwide^1,2^. For most cancer types, survival is significantly higher if the tumor is detected at an early stage^3,4^. Currently, mass population screening is applicable only for breast and cervical cancers and utilizes physical tests like mammography and cytology screens. Detection for other cancer types, done both *en masse* and in a low and affordable-resource setting, still poses a major challenge for the scientific and clinical communities^1^. In particular, a major hurdle is to identify reliable biomarkers for the detection of cancer at a presymptomatic stage. Detection at such an early stage would allow not only for improved survival but would also decrease treatment toxicity and provide an opportunity for providing personalized treatments.

Circulating cell-free DNA (cfDNA) has a lot of potential to become an effective biomarker for cancer diagnostics^5–8^. It has a short life span (16 min to 2.5 h), making it an efficient temporal indicator of biological processes in the body. With improvements in sequencing technologies and their costs becoming lower, cfDNA can also be a rapid and cheap biomarker. Being able to identify circulating tumor DNA (ctDNA) is also minimally invasive and can allow not only for cancer detection but also to monitor patients already diagnosed with cancer. To identify cancerous cells, their tissue of origin, cancer type, minimal residual disease, and other cancer features, current cfDNA sequencing technologies rely on the detection of somatic mutations^9^ and epigenetic marks, such as DNA methylation or histone modifications that can determine the cancerous tissue of origin^10,11^. For example, one study showed that cell-free DNA methylation patterns can be used for the accurate detection of early-stage breast cancer^12^. Furthermore, cfDNA has been used as a biomarker for treatment response and recurrence^13^. However, ctDNA still has many hurdles and caveats that need to be overcome^14^. Some of the major hurdles include: (1) Circulating cfDNA is found in fragment sizes ranging between 120 and 220 base pairs (bp), with most of the fragments being 167 bp^15^ in length, thus making it challenging to extract; (2) Tumor-derived DNA makes up around 0.4% of the total cfDNA (there is some variance between tumor types), thus needing extremely sensitive biomarkers that can detect the presence of cancerous cells; (3) Prior knowledge of specific mutations or methylation marks is required for targeted screening, leading to biased screening primarily of coding mutations which make up only a small fraction of cancer-associated mutations. (4) Somatic alterations in white blood cells can confound cfDNA mutations and epigenetic changes^16^; (5) The techniques used to detect methylation or histone marks from cfDNA are technologically complex and have low sensitivity and specificity, in particular in early stages^5,17,18^. To provide the most optimal cancer treatment, it needs to be diagnosed at early stages when the tumor is small (~5 mm in diameter). At these stages, the tumor produces very low levels of ctDNA that are difficult to detect using current methods^5^.

Nullomers are short DNA sequences (13–17 base pairs) that are absent from the human genome^19–21^. While the absence of nullomers could be due to chance, we and others have shown that a significant proportion of them is under negative selection pressure^20,21^, suggesting that they may have a deleterious effect on the genome. We have also shown that these sequences could be used as DNA “fingerprints” to identify specific human populations^21^. As nullomers generally do not exist in a human genome, their appearance due to mutagenesis followed by clonal expansion could be exploited as a diagnostic method for diseases associated with a mutational burden, such as cancer. Nullomers have been used for the detection of five different cancers using cell-free RNA^22^.

Here, we set out to test whether nullomers could be used as a diagnostic tool to detect cancer and various additional tumor features. Throughout this manuscript, we refer to nullomers uniquely found in multiple tumor genomes as *neomers* to distinguish them from the more general category. We first analyzed The Cancer Genome Atlas (TCGA^23^) database, finding that neomers created by somatic mutations are able to detect cancer subtypes with higher accuracy than leading methods^24^ as well as additional cancer features. Further analyses of cfDNA whole-genome sequencing (WGS) datasets found that these neomers can also be used to detect cancer subtypes. Using WGS on cfDNA from 465 individuals with ovarian or lung cancer and normal controls found an enrichment for neomers. Our neomer-based cancer detection models had area under the curve (AUC) of 0.93 for lung and 0.88 for ovarian cancers, and an AUC of 0.94 for lung and 0.93 for ovarian cancer at early stages. This is particularly noteworthy for ovarian cancer as currently there is no effective screening test^25^, with most women being diagnosed at later stages, IIIC or IV, where 5-year survival rates are 39% and 17%, respectively^26^. Finally, utilizing both promoter assays and a massively parallel reporter assay (MPRA), we show that neomers alter regulatory activity and can be used to detect cancer-associated mutations in gene regulatory elements. Combined, our results show that neomers can be used as a rapid, sensitive, specific, and simple cancer diagnostic tool and also aid in the identification of gene regulatory mutations associated with cancer.

## Materials and methods

### Computational characterization of nullomers

The GRCh38 reference assembly of the human genome was used throughout the study. Nullomer extraction was performed for kmer lengths up to 17 base pairs using the algorithm described in our previous work^21^. The reverse complement of a nullomer will also be a nullomer and throughout this manuscript when counting nullomers, the reverse complement of nullomer *i* was also considered separately, unless *i* is a palindrome. Substitutions and indels that were not present in healthy tissues were identified from WGS of tumor samples from 2577 individuals across 21 tissues were obtained from open access release data at https://docs.icgc-argo.org/docs/data-access/icgc-25k-data^23^. Recurrent nullomers (neomers) (*r*_*i*_) were annotated as those that resulted from substitutions or indels across two or more patients within a cancer type. When possible, *r*_*i*_ was chosen to get ~10,000 neomers from each tissue; otherwise, it was set to 2 (Data S1). Driver mutation-derived neomers were defined as nullomers detected from driver mutations and were identified using the catalog of driver mutations derived from the intOGen database^27^, for which we grouped driver mutations by tissue of origin.

### Classification of tumor tissue of origin using neomers

We trained a classifier to distinguish classified tissue of origin for a cancer sample based on observed neomers using the libSVM package for Julia^28^ with default parameters to train a support vector machine (SVM) classifier with a linear kernel. We used 10-fold cross-validation, whereby the classifier was evaluated on a held-out fraction of the data. The set of neomers for each cancer type was recalculated for each round to only include the patients in the training set.

### Supervised selection of nullomers

The MMR status of each biopsy sample was derived from ref. ^29^. The model was trained on neomers identified in MSI samples and the performance of the algorithm evaluated. Previous research has indicated that mismatch repair deficiency is associated with mutations at mononucleotide repeat tracts^30,31^. For the MSI versus the microsatellite stable (MSS) samples, we counted the number of neomers that contained either AAAAAAAA or TTTTTTTT repeats, since MSI cancers have been associated with mutations of polyA/T repeats^32^. The threshold for determining MSI or MSS was set as the harmonic mean of the maximum number of counts in the MSS set and the minimum number of counts in the MSI set. The POLE deficiency status of each biopsy sample was derived from ref. ^29^ and we used a similar strategy to that of MMR status, but instead counted neomers created through either a TCT > TAT or TCG > TTG mutation. Since the number of patients in each category was limited, we used a 5-fold cross validation. For ovarian cancer analyses, two sets of nullomers were used. From the nullomers of length *k* = 13 identified in ref. ^21^, we retained only the ones that could not be created by any of the single base-pair substitutions identified in gnomAD v2^33^. Similarly, we selected all nullomers of length *k* = 15 of order 1, i.e., nullomers that can only be created from the human reference genome through at least two insertions, deletions, or substitutions.

### cfDNA extraction and WGS

Lung and ovarian cancer samples, as well as age and gender-matched samples were purchased from ProteoGenex Inc. (Inglewood, CA, USA) and IndivuMed GmbH (Hamburg, Germany). In addition, cancer samples, and when available, healthy age and gender-matched samples, were also obtained from the labs of K. Syrigos (Sotiria Hospital, National and Kapodistrian University of Athens, School of Medicine, Athens, Greece), J. Chapman (UCSF, San Francisco, USA), J. Witte (UCSF, San Francisco, USA), and C. Maher (WashU, St. Louis, USA). The samples that included ancestry information were all Caucasian (Data S3). All samples were collected with informed consent. All human sample work was carried out under the approved UCSF human research protection program institutional review board protocol number 21-35920. cfDNA was extracted from 1 mL of plasma, following centrifugation at 4 °C at 34 × *g* for 3 min, to remove larger debris, using the QIAamp Circulating Nucleic Acid Kit (Qiagen). cfDNA was eluted in 50 μL of elution buffer and measured using the Qubit High-Sensitivity dsDNA kit, and validated for size distribution (160–180 bp) using an Agilent BioAnalyzer 2100 Sensitivity DNA chip. Up to 10 ng of cfDNA was used for sequencing library construction using the library preparation enzymatic fragmentation kit 2.0 (Twist Bioscience) adjusted with IDT’s xGen UDI-UMI 96 barcodes system (IDT) to replace the Twist universal adapter, and using KAPA HiFi polymerase instead of the polymerase provided by the kit. Libraries for lung, ovarian, and their respective controls were sequenced as PE150 using a NovaSeq 6000 S4 system (Illumina) leveraging UMIs to remove PCR duplicates, aiming for 5–10X coverage per multiplexed library, via Novogene. Samples were sequenced in batches of 96 (maximal UDI by the xGen kit) and were equally spread across multiple lanes. Each batch contains a consistent mixture of healthy controls and cancer samples.

### Neomer identification in cfDNA samples

To improve the detection of lung cancer mutations in cfDNA, we used neomers that are present in ≥2 patients rather than ≥3 as was used for the tissue-of-origin classifier. The FASTQ files were scanned for neomers by searching for exact matches to the 16-mers of interest. We disregarded any neomer that could arise due to a common germline variant (VAF > 1%). To reduce errors, only reads where the nullomer was found in both pairs were kept. To evaluate the ability to detect cancer from cfDNA, we first randomly split samples into training and validation sets. Prior to the split, samples were randomly removed from either the controls or patients to ensure that the two groups were of equal size. From the training set we determined a threshold, *t* = max (number of neomers in control samples from the training set)— 1. Samples in the validation set were then designated as having cancer if the number of neomers detected was ≥*t*. To calculate F1 scores, this procedure was repeated 100 times. For the downsampling analysis, each read pair in sample *i* was retained with a probability of *c/C*_*i*_, where *c* is the target coverage and *C*_*i*_ is the full coverage of the sample, i.e., sampling without replacement. For each value of *c* the downsampling was repeated 100 times.

### Classification model to detect cancer patients from cfDNA

Neomers were identified for each donor and combined as a neomer density × sample matrix, *d*_ij_ = *n*_ij_/*C*_j_, where *n*_ij_ is the count of neomer *i* in sample j and *C*_j_ is the sample coverage. Neomer-based classification models were generated to examine the ability of neomers to detect cancer for lung, colorectal, ovarian, pancreatic, and prostate cancers. We tested PCA and feature hashing to deal with the sparse nature of our dataset and used feature hashing with 8192 features as the best performing method. We evaluated five classification models; random forest, linear, and radial basis function SVMs and linear regression (LR) models with L1 and L2 regularization, each evaluated with a 10 × 10-nested cross-validation. Our benchmarks indicated LR with an L2 penalty as the best performing model across all cancer types. The results obtained were also examined regarding tumor staging for colorectal and lung cancers, in which we had sufficient sample sizes. The nested cross validation (CV) used a 10-fold inner CV to optimize model parameters and hyperparameters and a 10-fold outer CV to test the optimized classifier on data not used for parameter and hyperparameter optimization. This design yields an unbiased estimation of performance while allowing for full optimization of the model. Model parameters were optimized with respect to the balanced accuracy metric, as were hyperparameters across the inner CV. A grid search was used to determine the optimal hyperparameters. Model performance in the outer CV was evaluated using balanced accuracy, AUC, and other binary classification metrics. Choice of model type and of number of hashed features were made outside of the nested CV framework. In practice, the classifier performance tended to plateau or achieve a mild maximum with respect to the number of hashed features, so the number of hashed features chosen was sufficiently large to place the classifier in the performance plateau. No one model type consistently dominated the others in terms of performance, so the model type used was determined by the needs of the analysis and the different tradeoffs with respect to complexity and interpretability.

Fragmentomics-based classification models were generated to compare its performance against the neomer method on the lung and ovarian cancer datasets generated from this study. Paired-end sequencing reads were aligned to the GRCh38 reference genome using Burrows-Wheeler Aligner - Maximal Exact Matches (BWA-MEM).

Genome-wide fragmentation profiles for each sample (defined as the ratio of short to long fragments) were created using https://github.com/mhanbioinfo/fragmentomicsBEDPE, where GC-corrected counts were binned into 5 Mb windows. The fragmentation profile was then used in a stochastic gradient boosting model with 10-fold cross validation as described in ref. ^34^. Alternatively, the read coverage on each of the 39 autosomal arms was calculated and compared to the average coverage to generate coverage *z*-scores for each sample as described in ref. ^34^ and used as features for a stochastic gradient boosting model with 10-fold cross validation. For end motif analysis, duplicates from aligned reads were removed using Picard MarkDuplicates, and 167 basepair fragments were retained and converted to BEDPE format using samtools^35^. 256 tetranucleotide motifs at the breakpoints of 167 basepair fragments were used in a SVM and LR model using leave-one-out cross-validation as described in ref. ^36^.

### Promoter luciferase assays

Promoter sequences with and without the neomer (Data S4) were synthetically generated and cloned into the modified Promega promoter assay luciferase vector pGL4.11b (a gift from Dr. Rick Myers, HudsonAlpha) by BioMatik Inc. and Sanger sequence verified. LNCaP cells were plated at an initial density of 2 × 10^5^ cells/well in 24-well tissue culture plates and maintained in RPMI medium, 10% FBS supplemented with L-Glutamine and Penicillin/Streptomycin. Plasmids together with a Renilla expressing plasmid, pGL4.74 (Promega), at a ratio of 10:1 luciferase:Renilla were transfected using the X-tremeGENE™ HP DNA Transfection Reagent (Roche) using 1:4 ratio of DNA (μg) to reagent (μl). Seventy-two hours post-transfection, Luciferase and Renilla levels were measured using the Dual-Luciferase Reporter Assay System (Promega) following the manufacturer’s protocol using a GloMax Explorer Multimode Microplate Reader (Promega). Luciferase activity was normalized to Renilla levels and presented as Relative Luciferase Units (RLU). Statistical analysis was performed using Prism version 9.0.2 (GraphPad). All values were reported as means (AVG) and standard errors (SE). *p* < 0.05 were considered statistically significant.

### Massively parallel reporter assays

The lentivirus-based MPRA was performed following a previously published protocol^37^. An oligonucleotide library of 230 bp long fragments bearing: (1) 4609 sequences with and without a recurring neomer that was found in prostate cancer patients; (2) 100 scrambled control sequences randomly selected from the library. A pool of 230-nt oligos containing each of these 200-nt sequences flanked by 15-base pair primer sequences on either side was synthesized (Agilent Technologies), amplified, and cloned into the lentiMPRA plasmid. Briefly, amplified inserts were cloned upstream of a minimally active promoter (mP), 5’UTR barcode (BC), and EGFP reporter transcript. We aimed for ~100 BCs per sequence when constructing and extracting the plasmid library. Each insert was associated with a set of BCs using paired-end (PE150) sequencing on an Illumina NextSeq 500(see Gordon et al. Step 83^37^). Lentivirus was produced and titered and used to infect LNCaP cells at an MOI of 50 virus particles per cell. Three days post-infection, to remove non-integrating virus, DNA and RNA were extracted from the three replicates and used for library construction and multiplexing for sequencing. All MPRA-related sequencing was performed using an Illumina NextSeq 500 with either PE150 for the CRS-BC association library or PE15 for the DNA/RNA BC count portion of the protocol. MPRA analysis was carried out using MPRAflow^37^.

### Comparison to validated neoantigens

We downloaded a list of 1967 validated neoantigens from http://biopharm.zju.edu.cn/download.neoantigen/iedb_validated.zip. Requiring both predicted strong binding and a positive validation, provided 1700 neoantigens. To evaluate the enrichment of neoantigens corresponding to neomers, we assumed a hypergeometric distribution with 1700 draws from an urn with 188,659 white balls (total number of neomers) and 186,067,892 black balls (number of nullomers found with lower recurrency than what was required for neomers).

### Indel identification

Somatic indel calls were performed using three pipelines from four somatic variant callers. These included those from the Wellcome Sanger Institute, the DKFZ/EMBL, and the Broad Institute, which reported a somatic variant false discovery rate of 2.5%^23,30^.

### Statistics and reproducibility

Statistical tests are described in the context where they are used. All code is publicly available as described above for reproducibility of computational analyses.

## Results

### Annotation of mutations that lead to nullomers

**Fig. 1:**
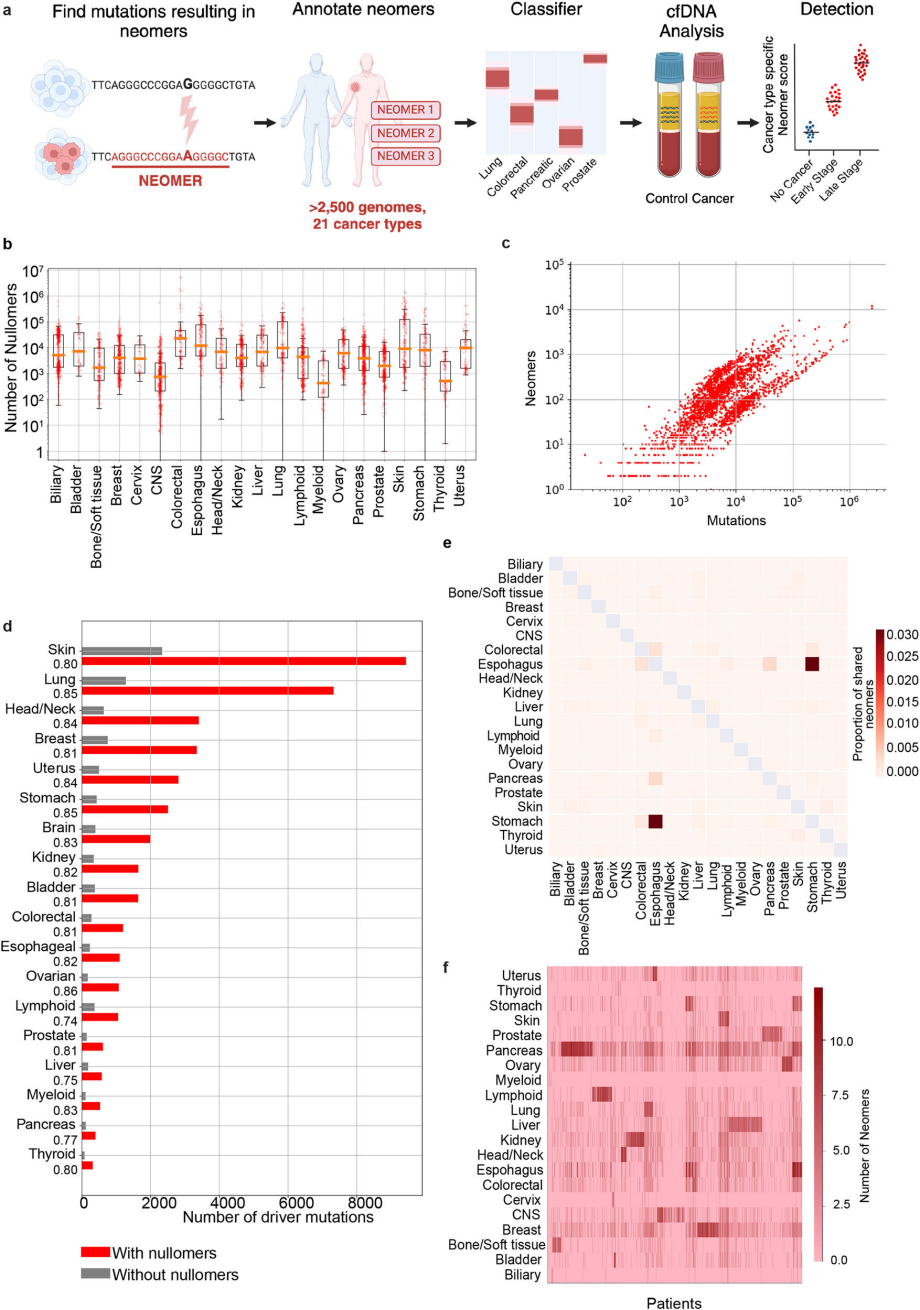
Neomers can detect cancer tissue of origin. **a** Schematic overview of neomer cancer diagnostic pipeline. Created in Biorender. Ahituv, N. (2025) https://BioRender.com/9sgstbs. **b–f** 2577 patients across 21 cancer types were used. **b** Number of neomers per patient sample across tissues. Each dot represents a patient sample. The boxplot displays the median as the central line. The bottom and top edges of the box represent the first quartile (25th percentile) and third quartile (75th percentile), respectively. The lower and upper whiskers extend to the smallest and largest data values that fall within 1.5 times the interquartile range above the 25th percentile and below the 75th percentile. **c** Number of neomers and the number of substitutions for 2577 patients (Spearman’s *ρ* = 0.75). **d** Identification of nullomers at driver mutation sites across cancer tissues. The majority of cancer driver mutations result in the resurfacing of nullomers. The proportion of mutations that cause the resurfacing of nullomers is indicated under each cancer type. **e** Heatmap showing the Jaccard index for the overlap of neomer sets associated with different cancer types. **f** Heatmap showing the occurrence of neomers across patients for each cancer type. Each row represents a cancer type and each column a patient. The intensity of the heatmap (log2-scale) shows the number of neomers for each tissue set.

As cancer is associated with a large number of somatic DNA mutations, we investigated if a subset of them could result in the resurfacing of nullomers (Fig. 1a). Using our previously characterized human nullomers^21^, we analyzed WGS results from 2577 patients across 21 cancer types from TCGA^38^ for resurfacing nullomers (Fig. S1A). We focused on 16 bp nullomers, as it is the shortest length where we detect a sufficient number of nullomers per patient, with the human reference genome having only 37.24% of all possible 16 mers. The majority of the 44,599,472 single-nucleotide substitutions that were tumor-unique give rise to multiple nullomers, allowing us to identify 213,164,038 resurfacing nullomers across all cancer types. Furthermore, we identified 2,470,091 nullomers resulting from short insertions and deletions (1–100 base pairs). The median number of nullomers created by each substitution was two, and for indels, four (Fig. S1B, C). On average, 58.29% of substitutions in a patient resulted in one or more nullomers, with 2.1% of the nullomers residing in coding regions. The median number of nullomers found across cancer patients was 9107 (Fig. 1b, c) and the number of nullomers was directly proportional to the number of mutations (Fig. S1D). As mutations were identified by comparisons to healthy tissues, germline variants of each individual were not included^38^.

To further prioritize nullomers that could be used as cancer biomarkers, we focused on the subset of nullomers that are recurrent, i.e., those found in more than one patient for a specific cancer type, termed hereafter as *neomers*. The number of neomers was proportional to the total number of mutations (Fig. 1c and Data S1). As both the number of patients per cancer type and the mutational load varied, the median number of neomers for each tissue type ranged from 0 to 98. Analysis of the most frequent neomers revealed several previously known cancer-associated driver mutations (Table 1). For example, some of the most recurrent coding neomers were the result of either the Gly12Asp, Gly12Val, or Gly12Cys missense mutation in the KRAS proto-oncogene GTPase (KRAS), which are known to make up 80% of cancer-associated KRAS mutations and lead to KRAS being constitutively active^39,40^. Although KRAS mutations have been associated with several cancer types, 190/215 (88%) of these mutations were found in pancreatic cancers. Several frequently occurring coding neomers were also found in other known cancer-associated genes, such as tumor protein p53 (*TP53*), B-Raf proto-oncogene serine/threonine kinase (*BRAF*), and phosphatidylinositol-4,5-bisphosphate 3-kinase catalytic subunit alpha (*PIK3CA*). The most frequent neomer was located in a noncoding region, within the telomerase reverse transcriptase (*TERT*) promoter, which is known to be associated with numerous cancer types^41^ and poor prognosis^42^. This mutation, called −124C > T or C228T, is extremely common in numerous cancer types^43^ and is thought to disrupt a G-quadruplex^44^ leading to the binding of GABP^45^, an ETS transcription factor, resulting in increased *TERT* expression. We found this mutation in 97 patients, with the highest incidence in glioblastoma (51%), fitting with its known high prevalence rate and diagnostic use for this cancer type^46^.

**Table 1:** Common cancer-associated neomers

Neomer	Locus	Coordinate (hg38)	Mutation	Name	Patient #	Cancer #
AGGGCCCGGA**A**GGGGC	*TERT*	chr5:1295113	G > A	−124C > T C228T	97	7
TCTTGCCTACGCCA**T**C	*KRAS*	chr12:25245350	C > T	c.35G > AGly12Asp	89	6
CTG**T**TGGCGTAGGCAA	*KRAS*	chr12:25245350	C > A	c.35G > TGly12Val	79	6
ACGCCACGA**G**CTCCAA	*KRAS*	chr12:25245351	C > G	c.34G > T Gly12Cys	47	1
GGTGC**A**TGTTTGTGCC	*TP53*	chr17:7673802	C > T	c.818G > A Arg273Pro	35	13
GTGGGGGCAG**T**GCCTC	*TP53*	chr17:7675088	C > T	c.524G > A Arg175His	28	11

Six of the most common neomers created by a single mutation. The nucleotide in bold font and underlined is the neomer causing mutation.

We also identified several neomers that are frequently created by different mutations (Table 2). Interestingly, some of these frequently recurrent neomers are created by different mutations, yet are predominantly found in one cancer. For example, GTTTTTCTCCTAGACC is found 41 times in skin cancer at 32 different loci while CTGGCAGTGAGCCACG is found 26 times in liver cancer across 23 loci. The majority (98%) of these frequent neomers reside in noncoding regions, and many of them reside in intronic regions (35%). For example, CGACGTTCTGCCCACT is found in 32 loci, primarily in pancreatic and stomach cancer. Of those loci, 21/32 (65.6%) were found in noncoding regions nearby pancreatic cancer-associated genes. These include, for example, the C-C motif chemokine ligand (*CCL4*)^47^, the POM121 transmembrane nucleoporin like 12 (*POM121L12*), which is commonly mutated in gastrointestinal cancers^48^ and the potassium voltage-gated channel modifier subfamily V member 1 (*KCNV1*) gene, where promoter hypermethylation has been associated with both pancreatic^49^ and esophageal cancer^50^.

**Table 2:** Frequent cancer-associated neomers

Neomer	Loci #	Patient #	Cancer #	Cancer type
CGACGTTCTGCCCACT	32	74	7	Mainly pancreatic and stomach
GTTTTTCTCCTAGACC	32	41	2	All but one in skin
CTGCAGTGGCGCAATA	30	30	11	33% of the neomers in colon cancer
TTATAGGGGTCCAGTG	25	26	1 (Five of the top frequently recurring neomers created by several different mutations)	Colorectal only
CTGGCAGTGAGCCACG	23	26	2	21 in liver, 5 esophagus

Five of the top frequently recurring neomers created by several different mutations.

To further validate that nullomers can detect cancer-associated mutations, we examined whether they are enriched in known cancer driver mutations. We annotated a cohort of driver mutations from 28 different cancer types^27^ for their overlap with 16 bp nullomers. In the pan-cancer analysis, we identified 19,594,212 nullomers resulting from 50,167 putative driver mutations (Fig. 1f and Data S2). For specific cancer types, we found that on average, 81% of driver mutations resulted in one or more nullomer, ranging between 63.88% and 86.50% in pancreatic and lung cancer, respectively (Data S2). The number of nullomers also varied by tissue type, ranging between 92 in thyroid and 9434 in skin (Fig. 1d). In general, driver mutations were 1.4-fold more likely to result in the creation of a nullomer (permutation test, *p*-value < 0.001). Taken together, our results suggest that a large subset of clinically relevant cancer mutations are associated with neomers, i.e., recurrent nullomers.

### Cancer subtype neomer classifier

We next set out to assess whether neomers can be used to distinguish between cancer types. We filtered neomers by keeping only those that appeared >=*r*_*i*_ times in specific cancer type *i* (Data S1). Comparison of the set of neomers associated with each cancer type revealed a small overlap, as indicated by the Jaccard index which is <0.04, suggesting that each cancer type has a distinct neomer signature (Fig. 1e). We also counted the number of times neomers are found in each patient, finding that patients are strongly enriched for only one set of cancer-specific neomers (Fig. 1f).

**Fig. 2:**
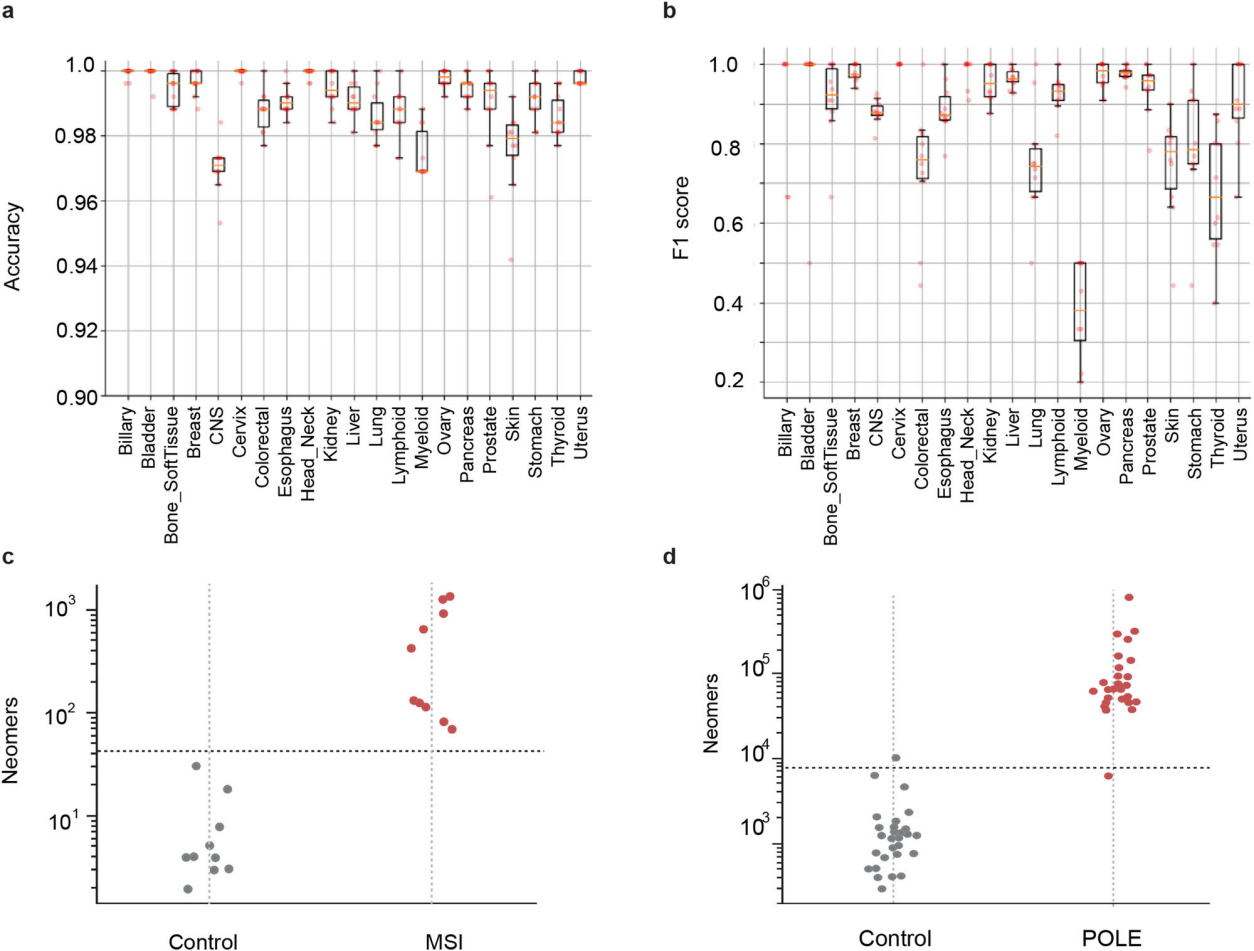
Neomers can distinguish cancer features. Boxplots of classifier accuracy (**a**) using an unsupervised classifier and F1 (**b**) score for the same classifier for each of the twenty-one cancer types. Box plot lines indicate lower, middle, and upper interquartile values. **c** Separation of 10 MSI and 10 MSS samples using a supervised selection of neomers. **d** Separation of 25 POLE proficient and 25 deficient samples using nullomers. In (**c**, **d**) the vertical line displays the harmonic mean.

We then tested if our annotated cancer-specific neomers can be used to classify tumor samples. We trained a SVM classifier to identify tumor type. The classifier takes as input a 21-dimensional vector indicating the number of neomers found for each cancer-specific set. For the classification model, we divide the samples into train and validation and test groups, and only filter the neomers and construct a classifier, based on the train samples. Evaluation using 10-fold cross-validation revealed that our classifier achieves both high sensitivity and specificity, with an F1 score of 0.92 and an accuracy of 0.99 (Fig. 2a, b). Performance was better than a recent deep learning model^24^ and also requires less computational resources to train as once neomers have been extracted, training and testing take only a few minutes.

### Neomers distinguish additional cancer features

We next tested whether a supervised feature selection approach, i.e., using neomers that are thought to be informative based on prior biological knowledge, would improve performance. For this approach, we initially utilized microsatellite unstable (MSI) and MSS cancers. MSI is associated with better cancer prognosis, increased benefits from surgery and higher sensitivity to immunotherapy, but with a lack of efficacy from adjuvant treatment^51^. Since MSI cancers are associated with mutations at polyA and polyT stretches^52^, we hypothesized that neomers containing these motifs would be able to effectively distinguish these two cancer types. We identified ten MSI samples from a cohort of 560 breast cancers^32,53,54^ and compared them to ten randomly selected MSS samples from the same cohort. We found that the polyA/T neomers were able to separate the two categories with an accuracy of 100% (Fig. 2c).

We next applied a similar strategy to distinguish patients with DNA polymerase epsilon catalytic subunit (POLE) deficiency, as these tumors are known to respond more favorably to immune checkpoint inhibitors^54^. We identified 25 patients from the TCGA dataset labeled as POLE deficient, and searched for neomers created through a TCT > TAT or TCG > TTG mutation, which are the most common types of mutations in this context^55,56^. Comparing against POLE proficient tumors, we found that the number of neomers identified for each group have very little overlap (Fig. 2d), and the classifier achieved an accuracy of 96%.

### Neomers detect early-stage lung cancer

**Fig. 3:**
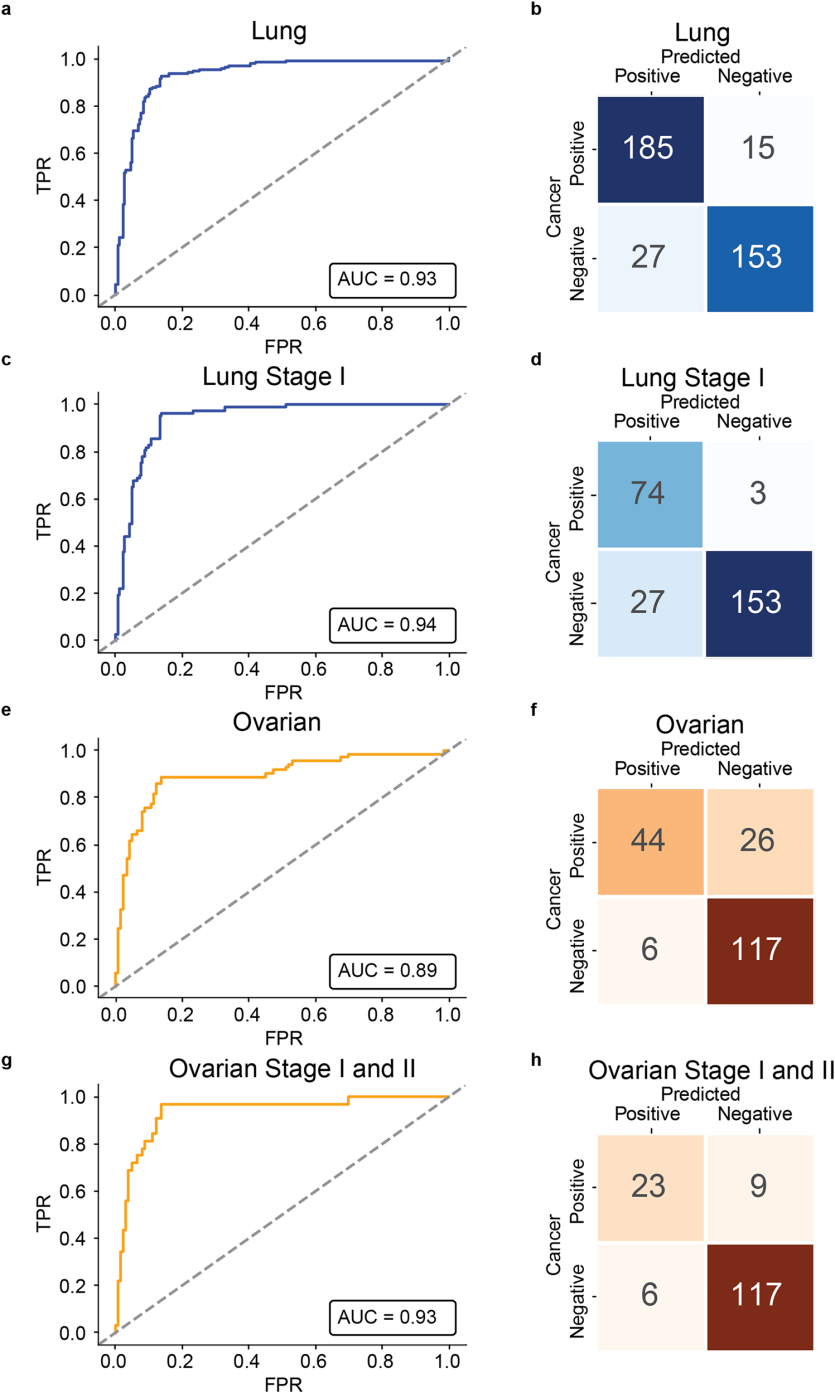
Neomers can detect lung and ovarian cancer from cfDNA. Receiver operator characteristics (ROC) curve (**a**) and confusion matrix (**b**) for the neomer based classifier for cfDNA from 200 lung cancer patients and 180 controls. ROC curve (**c**) and confusion matrix (**d**) from 77 stage I lung cancer cases and 180 controls. ROC curve (**e**) and confusion matrix (**f**) for ovarian cancer from 70 cases and 123 controls. ROC curve (**g**) and confusion matrix (**h**) for 24 stage I and 8 stage II ovarian cancer and 123 controls.

We next tested whether neomers could be used to diagnose cancer in cfDNA. We first used lung cancer as our test case, due to having an ample amount of cancer-associated neomers (Data S1), difficulty to diagnose using current techniques, with early stages being mostly asymptomatic^55,57^, and being the leading cause of cancer-associated mortality worldwide^55^. We assembled a cohort of cfDNA from the plasma of 381 individuals. These included 200 lung cancer patients from all stages with a strong emphasis on early stages: 77 stage I (38% of cancer cases), 36 stage II (18%), 34 stage III (17%), and 53 stage IV (27%). The majority of our cases were adenocarcinoma (50.2% of cancer samples), followed by squamous cell carcinoma (37.8%) (Data S3). The median age was 64 ± 7.8 years old, with patients as young as 42 years old. We also had 180 controls, considered cancer free (e.g., “healthy”, no cancer of any kind detected >2 years after sampling) with a median age of 58 ± 8.1 years and 90% fitting the criteria for the United States Preventive Services Task Force (USPTF) age interval for early screening for lung cancer. Of the healthy individuals, 21% were active smokers (37.2% were noted as unknown), while 64.2% of the cancer patients were noted as active or past smokers (complying with USPTF guidelines for lung cancer screening) (additional information on the cohort is available in Data S3). Samples were paired-end sequenced at a genomic depth of 5-10× coverage (*C*) and analyzed for neomers. For each sample and each neomer, all occurrences of the neomer were extracted from the FASTQ file, and the resulting neomer count was normalized by dividing by the sample read depth, forming a neomer density measure. Putting all the neomer densities into a table with sample rows and neomer columns yields a neomer density matrix (NDM). The NDM with an added column for the sample status forms the input for our classification models. To reduce the impact of sequencing errors, we only considered neomers that were found on both read-pairs from a single pair-end read, resulting in 18,090–55,409 read-pairs per sample. To further avoid spurious neomers, only neomers that overlapped at least two read-pairs and overlapped the human genome were used for subsequent analyses. Of note, an important computational advantage compared to conventional mutation calling pipelines is that only a single pass is made across the reads to identify those containing neomers, and only those reads are aligned to the reference genome. Using these data, we trained an LR classifier, achieving an AUC score of 0.93 using 10-fold cross validation and a sensitivity of 92.5% (specificity of 85%) (Fig. 3a, b). Importantly, our classifier also performed well for early stages, with an AUC of 0.94 for stage I and sensitivity of 96.1% (specificity of 85%) (Fig. 3c, d). To showcase the ability of the method to recognize different cancer types, we tested the performance of the classifier on lung cancer samples when utilizing the ovarian neomer list. The performance for all models is clearly lower than the performance of the optimal model trained on lung cancer neomers, indicating the ability of the classifier to discriminate between cancer types—a sample will be given a much higher chance of being from a cancer patient when using the corresponding cancer neomer list (Fig. S3). Finally, by carrying out a downsampling analysis, we find that already at 3x coverage we can obtain similar accuracy (Fig. S2). In summary, these data suggest that our neomer lung cancer profile can effectively detect lung cancer from cfDNA with high accuracy, including early stages.

### Neomers detect early-stage ovarian cancer

We next set out to test how our neomer classifier performs with a low amount of tumor-associated neomers. We focused on ovarian cancer which only has 12,788 associated neomers (amongst our analyzed TCGA WGS datasets) (Data S1). In addition, as ovarian cancer does not have an effective screening test^25^, most women are diagnosed at stages IIIC and IV, when 5-year survival rates are 39% and 17%, respectively^26^, reflecting a pressing need for early detection methods. We obtained plasma samples and generated cfDNA from 70 patients (Data S3) with a median age of 57 ± 16.1 years. Our analyzed cohort comprises 45.7% early stage samples (24 stage I and 8 stage II), and the rest from late stage (34 stage III and 4 stage IV). Samples were similarly paired-end sequenced at a genomic depth of 5–10× coverage (*C*) and analyzed for neomers. As controls, we used female donors from our control donor group, totaling 123 control cfDNA samples with a median age of 58 ± 7.9 years (additional information on this cohort is provided in Data S3). Using the same classifier as before, we obtained an AUC of 0.89 and a sensitivity of 62.86% with a specificity of 95.12% for all stages (Fig. 3e, f). Importantly, for early stages (stage I and II), we obtained an AUC of 0.93 and sensitivity of 71.9% with specificity of 95.12% (Fig. 3g, h). In addition, we carried out a downsampling analysis and we find that already at 3× coverage we can obtain high accuracy (Fig. S2). Combined, these results suggest that neomers can detect cancer even with a low tumor mutation burden and, more importantly, could potentially be used to diagnose early-stage ovarian cancer for which there is currently no available detection method. We also compared our approach to fragmentomics-based algorithms. We found that after implementing a fragmentomics-based stochastic gradient boosting model on our dataset, using 10-fold cross-validation, we obtain AUCs of 0.83 for lung cancer and 0.87 for ovarian cancer (Fig. S6A, B)^34^. Additionally, we extracted 256 end motifs from all 167 basepair fragments across the genome and used a SVM and LR model to differentiate healthy from cancer samples^36^. This model had an AUC of 0.75 and 0.81, respectively for lung cancer and AUC of 0.93 and 0.94 for ovarian cancer (Fig. S6C, D). Finally, we also implemented a stochastic gradient boosting model for lung cancer detection using coverage *Z*-scores derived from 39 autosomal arms (AUC 0.87 for lung cancer, AUC 0.93 for ovarian cancer) (Fig. S6E, F)^34^. Our analyses show that the neomer-based method performs comparably or better than existing fragmentomic approaches for cancer detection using the same cohort and preprocessing pipeline. Notably, it achieved higher or similar AUCs across models for both lung and ovarian cancer, highlighting its robustness and potential clinical utility.

### Neomers alter promoter activity

**Fig. 4:**
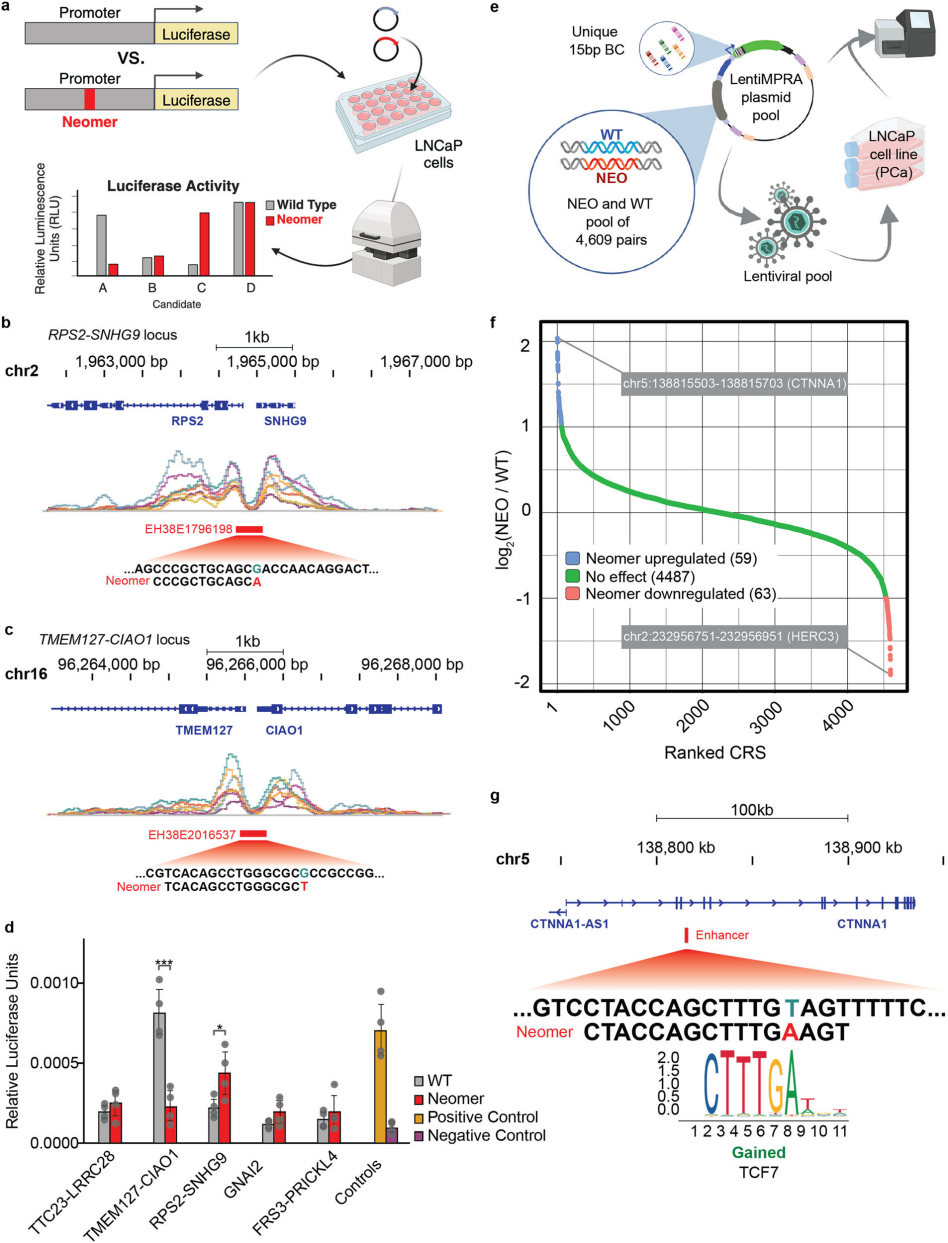
Neomers alter the activity of gene regulatory elements. **a** Schematic describing the luciferase reporter assay. Created in Biorender. Ahituv, N. (2025) https://biorender.com/08yc3gj. Integrative Genomics Viewer track snapshot showing the location of a prostate cancer-associated neomer in the promoters of *RPS2*-*SNHG9* (**b**) and *TMEM127*-*CIAO1* (**c**). The top track shows the gene location (GENCODE V36), beneath it the ENCODE layered H3K27ac marks in this region, below that the promoter location (red line) according to ENCODE cCRE annotations and the most lower track shows the neomer sequence. **d** Relative luciferase units from a luciferase promoter assay (*n* = 4 per condition) containing either the wild-type (WT) or nullomer variant. Transfection efficiency was normalized using Renilla luciferase and significance is calculated using a two-way ANOVA with multiple testing and Šidák correction. **e** Schematic showing the lentiMPRA workflow testing 4906 wild type (WT) and neomer (NEO) sequences. **f** The regulatory impact of 4609 neomer creating mutations, presented as log2 fold change of neomers containing sequence over wild type sequence (*x*-axis) and ordered from highest to lowest (*y*-axis). **g** A neomer in the *CTNNA1* locus had the highest activating score and is postulated to lead to a gain of a TCF7 motif, as predicted by FIMO^71^.

Only a small number of mutations in gene regulatory elements have been found to be associated with cancer^38,58,59^. As our results show that driver mutations are enriched for neomers (Fig. 1d) and the majority of our neomers (98%) lie in noncoding regions, we hypothesized that neomer-creating mutations in noncoding regions could be used to detect cancer-associated mutations in gene regulatory elements that have a functional consequence. Of note, the top patient recurrent neomer was in the *TERT* promoter (Table 1), which is associated with numerous cancers^59^. We chose to focus on prostate cancer since it is associated with a relatively small number of neomers (*N* = 4621; median per patient = 29.5), allowing us to characterize all the detected neomers for this cancer via MPRA in the following section. Moreover, 80–90% of prostate cancer is dependent on androgen receptor (AR) signaling^60^, allowing us to use LNCaP-FGC cells, which are androgen-dependent, for our reporter assays. We initially set out to test neomers found in promoters for luciferase reporter assays (Fig. 4a) using the following criteria: (i) neomers that reside in a promoter based on ENCODE annotations^61^; (ii) the gene regulated by the promoter is associated with prostate cancer. Our list (Data S4) included neomers in: (1) a promoter between two divergent genes, *RPS2* and the lncRNA gene *SNHG9* (Fig. 4b), both of which are overexpressed in prostate cancer^62^; (2) a promoter between two divergent genes, *TMEM127* and *CIAO1* (Fig. 4c), with the former being downregulated in prostate cancer^63^; (3) a promoter between two divergent genes, *TTC23* and *LRRC28*, with the former showing aberrant splicing that relates to therapy resistance in prostate cancer cells^64^; (4) The promoter of *GNAI2*, a protein that interacts with *CXCR5*, which positively correlates with prostate cancer progression^65^; (5) A promoter between two divergent genes, *PRICKLE4* and *FRS3*, with the latter thought to affect malignant but not benign prostate cells^66^.

We cloned the promoter sequences with and without the neomer into a luciferase promoter assay vector and compared their activity in LNCaP cells (Fig. 4a). Two out of the five assayed promoters showed a significant effect on reporter activity between the neomer and wild type allele (Fig. 4d). For the *RPS2*-*SNHG9* promoter, the neomer led to significantly increased activity, in line with this gene being overexpressed in cancer^62^. For the *TMEM127*-*CIAO1* promoter, the neomer completely abolished activity, fitting with its observed downregulation in prostate cancer^63^. Combined, our experimental results show that neomers could have a significant effect on promoter activity and could potentially be used to identify cancer-associated gene regulatory mutations.

### Neomers alter enhancer activity

We next set out to test in a high-throughput manner whether neomers could affect the function of active gene regulatory elements, such as promoters and enhancers. We annotated all the prostate-associated neomers that reside in noncoding regions, selected for those that were recurrent in at least three patients (to increase likelihood to be cancer-associated and reduce sequences to be assayed due to MPRA limitations), obtaining 4609 loci and generated a lentivirus-based MPRA (lentiMPRA) to test the regulatory activity with and without the neomer of all these sequences (Fig. 4e). Two hundred base pair sequences, where the position of the neomer is used as a center, were synthesized and cloned upstream of a minimal promoter followed by a GFP reporter gene. Lentivirus was generated and the library was infected using three technical replicates into LNCaP cells and RNA and DNA barcodes were sequenced to determine regulatory activity, as previously described^37^. We observed a good correlation between RNA/DNA ratios (Pearson 0.72-0.77; Fig. S4A) as well as for DNA and RNA barcodes separately (Fig. S4B, C) across all three technical replicates. Out of the 4609 loci, 567 showed significant regulatory activity due to the neomer (RNA/DNA ratio ≥ 1.5). Amongst them, 94 neomers and wild type sequence pairs showed significant (*p*-value ≤ 0.05, U-Mann–Whitney test) differential enhancer activity, with 48 having increased activity due to the neomer (NEO/WT ≥ 2) and 46 decreased activity (NEO/WT ≤ 0.5) (Fig. 4F and Data S5).

The neomer that showed the highest level of increased activity compared to reference (4.09-fold) is located in an intron of the catenin alpha 1 (*CTNNA1*) gene. This gene is a core member of the cadherin/catenin complex and is involved in the regulation of the Wnt/beta-catenin pathway, which has been widely studied in cancer^67^, including prostate cancer^68^, and is the target of several therapies^69,70^. TFBS analysis of this neomer using FIMO^71^ and JASPAR^72^ found that it leads to a gain of a TCF7/TCFL1 motif (MA0769.2) (Fig. 4G), which is known to play a role in prostate cancer malignancy^73,74^. A neomer residing in the third intron of the HECT and RLD domain containing E3 ubiquitin protein ligase (*HERC3*) gene led to the lowest downregulation of reporter activity (2.7-fold; Fig. 4F). HERC3 is an AR target and its dysregulation has been associated with epigenetic modulations and poor outcome in prostate cancer^75^. In addition, for colorectal cancer, this gene was found to be downregulated, is known to inhibit metastasis and is associated with overall lower survival^74^. Taken together, our MPRA results suggest that neomers could be utilized to identify gene regulatory mutations in cancer.

## Discussion

Cancer is a DNA mutation-associated disease. Here, we show that by analyzing cancer WGS, both from tumors and cfDNA, we can find cancer-associated DNA mutations that lead to the generation of neomers, short sequences that are predominantly absent from genomes of healthy individuals. Further analyses of these sets of neomers show that they can be used not only to classify cancer tissue of origin, but also to identify additional cancer features, such as MSI or POLE deficiency, with high accuracy. Analysis of cfDNA WGS generated from lung and ovarian cancer patients finds that neomers could be used to detect cancer, including in early stages, with high specificity and sensitivity. This is particularly important for ovarian cancer, for which there is currently no effective screening test^25^ with most women being diagnosed at later stages when survival rates are low^26^. We were able to detect early-stage (stage I and II) cancer with an AUC of 0.93. Finally, using promoter assays and MPRA, we show that neomers can be used to detect cancer-associated mutations that have a functional effect on regulatory element activity.

We utilized 2577 patients from 21 different cancerous tissues to develop a cancer tissue of origin classifier. Overall, we observed almost no overlap between neomers found in different cancer types. This allowed us to detect cancer tissue of origin with extremely high specificity and accuracy (F1 score of 0.92 and an accuracy of 0.99), performing better than recent deep learning models^24^. In general, the classifier has better performance for cancer types with more patients and high mutation burden (Fig. 2a, b). Our analyses also showed that other than tissue origin, nullomers can also be used to detect additional cancer features. Future work that will utilize additional cases and metadata would allow us to test whether nullomers and neomers can diagnose additional tumor features and also detect other cancer characteristics such as chance of recurrence, predict and/or monitor response to treatment, disease-free survival, and overall survival.

For cfDNA, despite various known challenges, including fragmentation and low levels of ctDNA, we obtained high AUC scores, and our results for lung cancer compare favorably with a recent study based on metabolite profiling^76^. Lung cancer, being one of five cancer types with a widespread approved screening option, utilizes a low-dosage CT scan (LDCT) for populations at risk (age, smoking, etc.). LDCT’s sensitivity was 93%, specificity was 84% when taking all stages together^77–80^. Recently, the employment of a sequencing-based fragmentomics assay followed by LDCT provided a stage-weighted overall sensitivity of 84% (95% CI: 78–90%)^81^. For ovarian cancer, which is associated with a low tumor mutation burden and has hitherto been extremely challenging to detect at early stages, we managed to detect, even with low sample numbers (*N* = 70), both late and more importantly early stages (stages I and II), obtaining an AUC of 0.93 and sensitivity of 71.9% with specificity of 94.44%. Our higher AUC scores in early stages could be due to larger sample sizes at this stage and are in line with other publications that find higher scores in these early stages compared to late stages^34,82,83^.

It is worth noting that our classifier did not benefit from removing neomers that can arise due to common germline variants. Nevertheless, we expect that knowing the donor’s ancestry could allow us to further improve the classifier. However, this would require more detailed metadata than was available in the present study. Since the number of neomers increases with the number of tumor samples profiled, obtaining additional WGS datasets from tumors and matched controls would also be beneficial in improving the ability of our classifier to diagnose both these and other cancers. Additional testing across multiple cancer types and in a larger number of samples is required to determine if the specificity that we obtained when using neomers to classify ICGC primary tumor samples can be maintained. We assume that, as cfDNA only contains a small fraction of tumor DNA, the signal will be much weaker.

Our cfDNA detection approach has several advantages over current methods: (1) Detecting short DNA sequences that are enriched in cancer samples provides an easy-to-use diagnostic that could allow detection from low amounts of ctDNA. In addition to sequencing-based assays, alternate techniques could potentially be used, such as CRISPR-based diagnostic tools^84^, that can also allow the testing of thousands of sequences in parallel^85^. In addition, with neomer-based diagnostics potentially not needing large amounts of starting material, cfDNA could be collected from urine, sputum, saliva, or other bodily fluids, which were shown to be a viable but reduced source of cfDNA^86,87^. (2) Our WGS approach is applicable to more sparsely sequenced samples. As also shown in our downsampling analysis, we obtain similar accuracy for both lung and ovarian cancer already at 3× coverage (Fig. S2). It is worth noting that this study was performed using standard Illumina sequencing. The use of sequencing technologies that provide longer reads^88^ and/or improved accuracy^89^ could further improve the ability of neomers to detect cancer. (3) As our diagnostic is based on neomer detection followed by genome alignments of nullomer encompassing reads, it is extremely effective from a computational standpoint. 4) Neomers could easily be combined with other sequence or analyte-based cancer biomarkers and risk factors to improve the diagnostic positive predictive value. For example, it was shown that combining a blood test that detects both protein biomarkers and DNA mutations, along with positron emission tomography-computed tomography (PET-CT), could detect multiple cancers^90^. In another example, the use of cfDNA fragmentation patterns combined with CT imaging, clinical risk factors, and serum levels of carcinoembryonic antigen significantly increased the ability to diagnose lung cancer^34^. Adding neomers to other known cancer-associated mutations (coding, regulatory, CNVs, etc.) in the screening of cfDNA could also increase sensitivity and specificity. This should be relatively straightforward, as our neomer detection method is based on standard sequencing technologies. In summary, coupling neomer-based diagnostics to existing cancer biomarkers and risk factors could improve the power to detect various cancer subtypes.

As nullomers/neomers do not exist in the human genome they could also be exceptional candidates as suicide genes, as shown previously for three nullomers^91^, or for neoantigens, to be targeted via immunotherapy. Previous work has shown that minimal absent words, short sequences that are absent from a genome or proteome, could be used to identify phosphorylation sites of high confidence, some of which could be associated with cancer^92^. Analysis of the Immune Epitope Database of validated antigens^93^ found that 13 of the recurrent coding neomers can create neoantigens with predicted strong binding levels that were subsequently validated (Data S6). From the 1700 neoantigens with strong binding levels, only 1.72 (*p*-value < 1e-8, hypergeometric test) is expected to correspond to a neomer, suggesting that missense mutations also resulting in neomers are 7-fold more likely to also generate strongly binding neoantigens.

Neomers can be used as a tool to identify cancer-associated gene regulatory mutations. Amongst the 210 prostate cancer promoter neomers, we selected five promoters and found that two of them significantly affected promoter activity due to the neomer. Their difference in activity was in line with the gene’s expression change in prostate cancer, with *RPS2*-*SNHG9* having increased activity, fitting with *RPS2* overexpression in prostate cancer^94^ and *TMEM127*-*CIAO1* abolishing activity, in line with *TMEM127* observed downregulation in cancer^63^. Our MPRA library of 4609 neomer-causing mutations in enhancers revealed that 2.6% can change gene expression by >1.5-fold, suggesting that a subset of these mutations could have important functional consequences in cancer. While we obtained good correlations between our three MPRA replicates, MPRA has some caveats that should be noted: (1) testing genes outside of their genomic context; (2) not being able to assign the target gene; (3) utilizing a minimal promoter instead of the target promoter; (4) using cell lines and one prostate cancer cell type. Nonetheless, understanding tumor onset and its transition to a metastatic cancer has been limited mostly to coding genes or their pre-determined regulatory sequences^95,96^. With the vast majority of mutations falling into non-coding regions, making them much more challenging to interpret, novel approaches are required for identifying mutations that have functional consequences. Here, we show that by prioritizing mutations that result in neomers and utilizing MPRA, it is possible to shortlist candidates that can have a phenotypic impact. This approach can be applied to any cancer type and thus holds the potential to expand our catalog of functionally relevant cancer driver non-coding mutations.

In summary, we show that neomers can provide a powerful tool for cancer diagnosis. As they can easily be detected via sequence or CRISPR-based tools, it should be straightforward to integrate them in current routine cancer diagnostic tests and their use could increase the sensitivity and specificity of these tests. Combining neomer-based screening with clinical characteristics and additional diagnostic tools and features could increase the positive predictive value. In addition, as cfDNA could also be isolated from urine and saliva, and detection of these sequences only requires a relatively small amount of DNA, neomer-based diagnosis could be carried out in a non-invasive manner. The utilization of neomers in the clinic will require additional work. This includes standardization of the collection and processing of patient samples, clinical trials that use neomers for cancer detection in a Clinical Laboratory Improvement Amendments certified lab and further refinement and standardization of our algorithms. Our work also suggests that neomers could be used to highlight cancer-associated gene regulatory mutations, which have been difficult to identify. Further high-throughput characterization of these mutations could allow the detection of bona fide cancer-associated functional regulatory mutations that could be used for diagnosis and treatment.

### Reporting summary

Further information on research design is available in the Nature Portfolio Reporting Summary linked to this article.

## Supplementary information

Supplementary Information

Description of Additional Supplementary files

Supplemental Data 1-6

Reporting summary

## Data Availability

The source data for Fig. 1 is found in Data S1, for Fig. 3 in Data S3, and for Fig. 4 in Data S5. MPRA sequencing data was deposited in the NCBI short read archive (SRA) with accession code PRJNA917083. ICGC samples are publicly available and can be accessed from https://docs.icgc-argo.org/docs/data-access/icgc-25k-data. Due to patient data confidentiality, WGS sequencing data is only available upon request. For WGS sequencing, contact Nadav Ahituv.

## Code availability

We generated an easy-to-use software package that enables performing nullomer cancer analyses from sequence-based datasets^97^. The package is composed of six functions: (1) EnumerateNullomers, which extracts all nullomers of specified kmer lengths in a FASTA sample; (2) ExtractMutationNullomers, which finds all mutations that cause the resurfacing of a list of nullomers; (3) IdentifyRecurrentNullomers, which identifies nullomers that recur in a dataset through mutagenesis; (4) FindAlmostNullomers, which identifies the positions that can create a list of nullomers genome-wide for every possible substitution and single base-pair insertion and deletion; (5) FindNullomerVariants, which removes nullomers that are likely to result from common variants in a user specified variant VCF file; (6) FindDNANullomersFromReads, which performs the identification of nullomers in raw read samples. The package can be found at 10.5281/zenodo.1091017397 and the documentation on Read the Docs (https://nullomerator.readthedocs.io/en/latest/#) provides in-depth details on how to use the software.
