# Supplementary material for: Leveraging sequences missing from the human genome to diagnose cancer
Source: Commun Med (Lond). 2025 Aug 21;5:363. doi: 10.1038/s43856-025-01067-3 (PMC12371106; doi:10.1038/s43856-025-01067-3)
Supplement: Supplementary file 1 — Supplementary Information [file 43856_2025_1067_MOESM1_ESM.pdf]

Leveraging sequences missing from the human genome to diagnose cancer

Ilias Georgakopoulos-Soares, Ofer Yizhar Barnea, Ioannis Mouratidis, Candace S.Y. Chan, Michail Patsakis, Akshatha Nayak, Rachael Bradley, Mayank Mahajan, Jasmine Sims, Dianne Laboy Cintron, Ryder Easterlin, Julia S. Kim, Emmalyn Chen, Geovanni Pineda, Guillermo E. Parada, John S. Witte, Christopher A. Maher, Felix Feng, Ioannis Vathiotis, Nikolaos Syrigos, Emmanouil Panagiotou, Andriani Charpidou, Konstantinos Syrigos, Jocelyn Chapman, Mark Kvale, Martin Hemberg, Nadav Ahituv

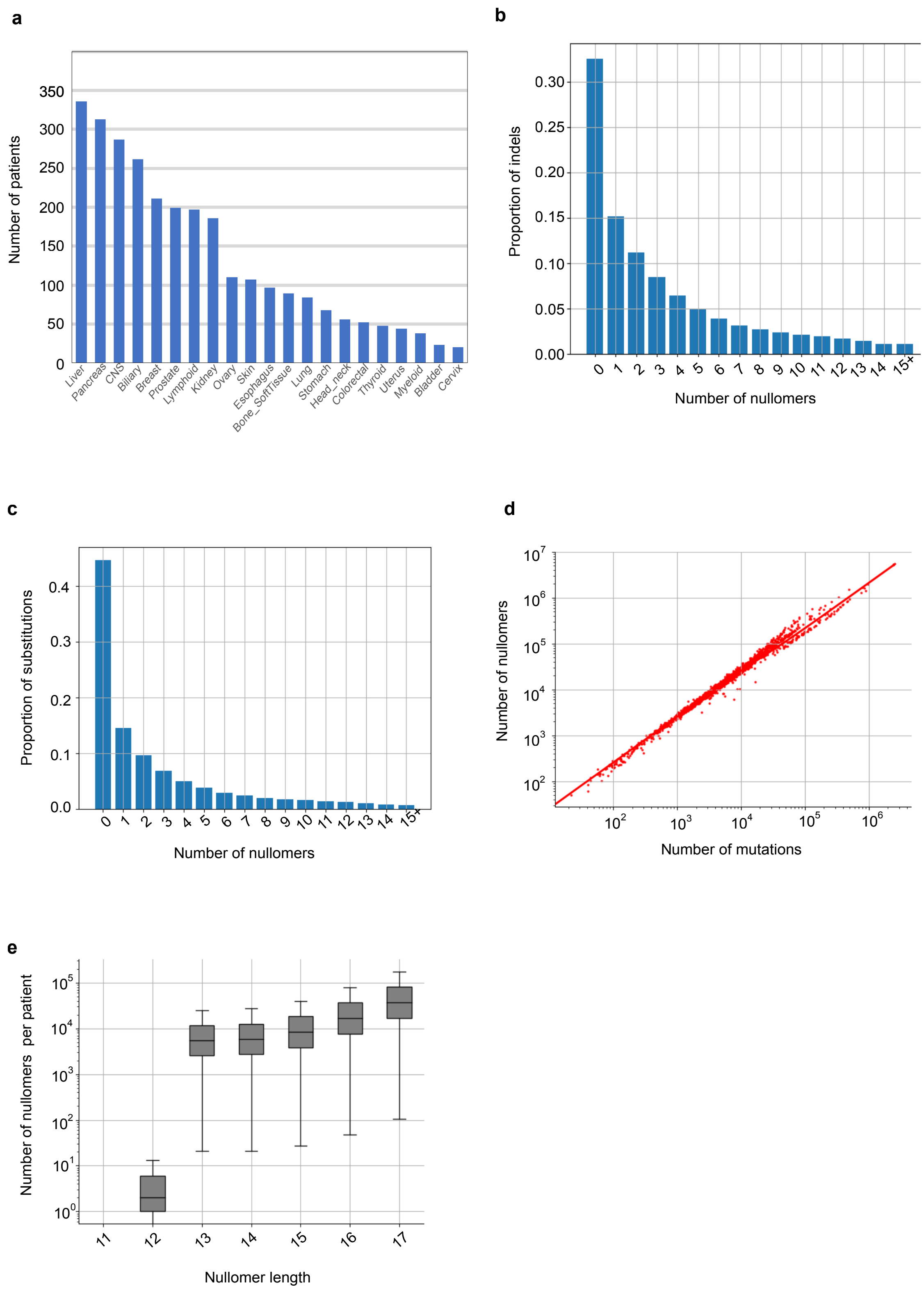

Supplemental Figure 1. Nullomer characterization. a. Number of patients per cancer tissue. **b-c.** Number of nullomers resurfaced due to indels or substitutions observed for each tumor sample (per patient) . **d.** Association between number of mutations and number of nullomers observed. **e.** Number of nullomers of different lengths observed per patient.

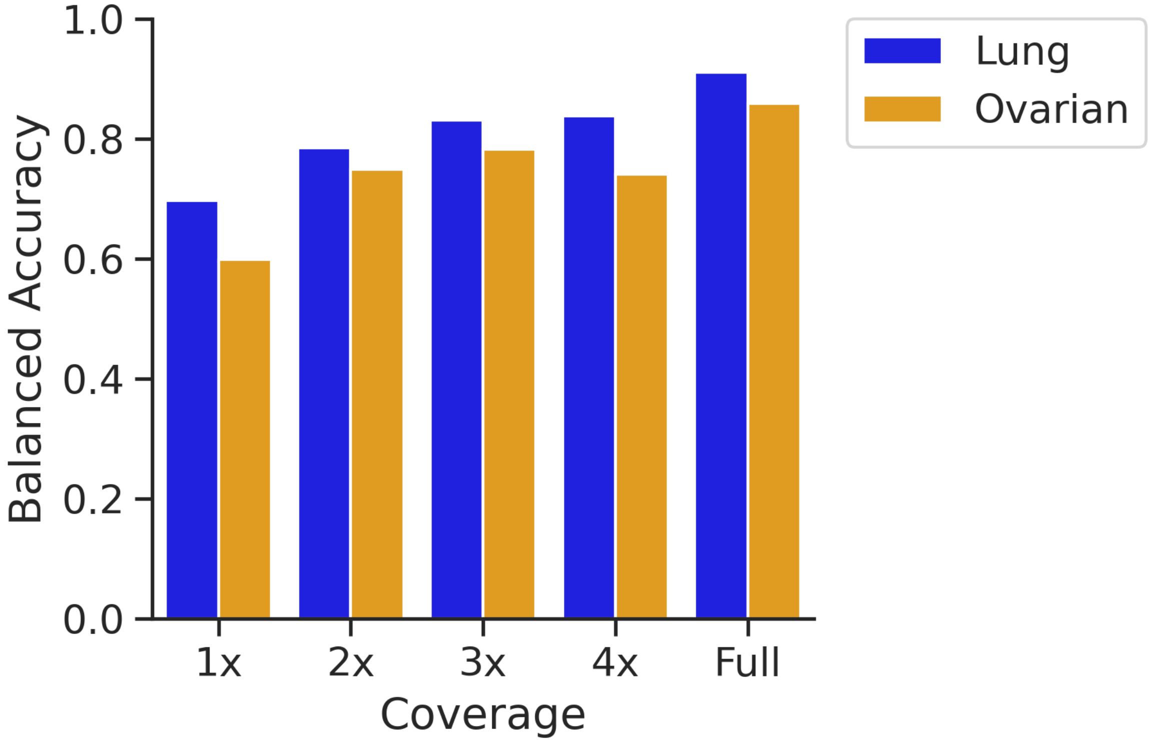

Supplemental Figure 2. Balanced accuracy of lung or ovarian cancer neomer-based assay using different levels of sequencing coverage.

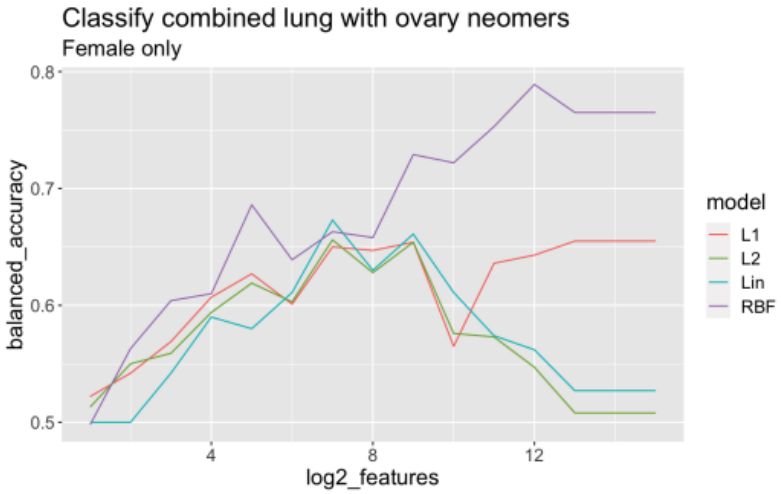

Supplementary Figure 3. Performance of the classifier on lung cancer samples, when utilizing the ovarian neomer list.

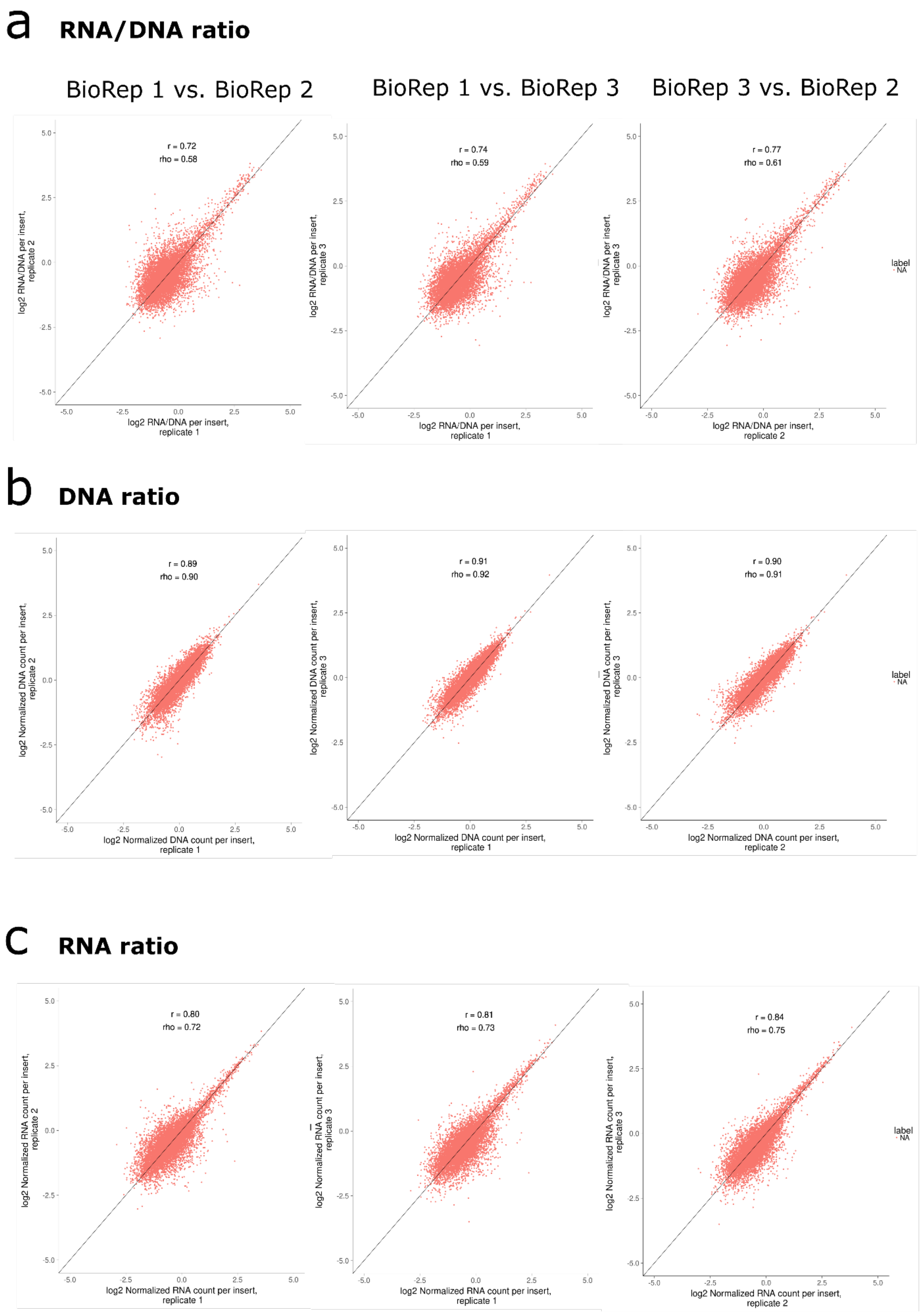

Supplemental Figure 4. Correlation between replicates in Prostate MPRA. a-c, All libraries sequences number of normalized reads across all barcodes correlation between the three biological replicates, as calculated for the RNA/DNA ratio **a.** DNA barcodes alone **b.** and RNA barcode alone **c**.

a

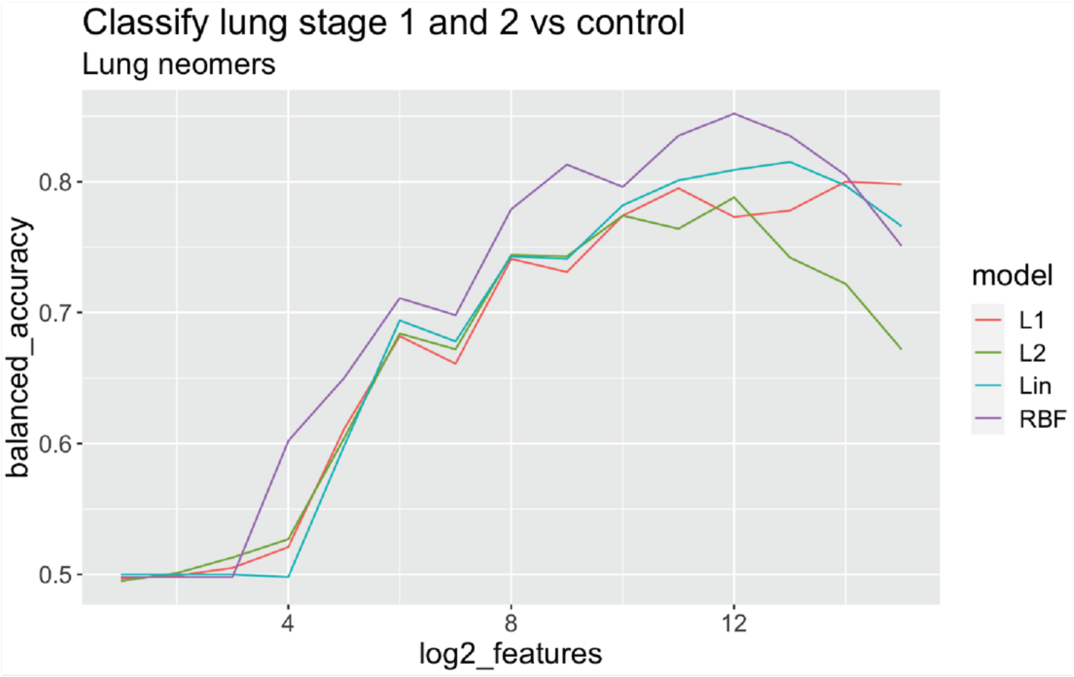

b

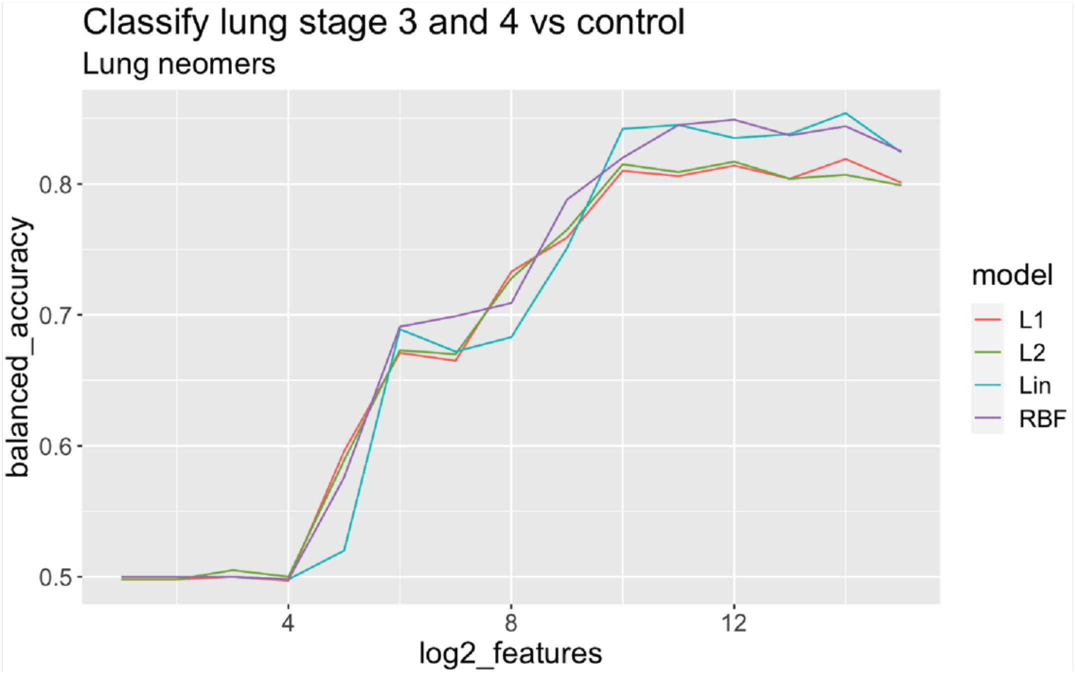

Supplemental Figure 5. Classification performance on lung cancer separated between **a.** stage I + II and **b.** stage III + IV. Feature hashing was used to preprocess the data and the balanced accuracy is reported as a function of the number of hashed features. Models reported are logistic regression with L1 regularization and L2 regularization and linear (lin) and radial basis function (RBF) support vector machines.

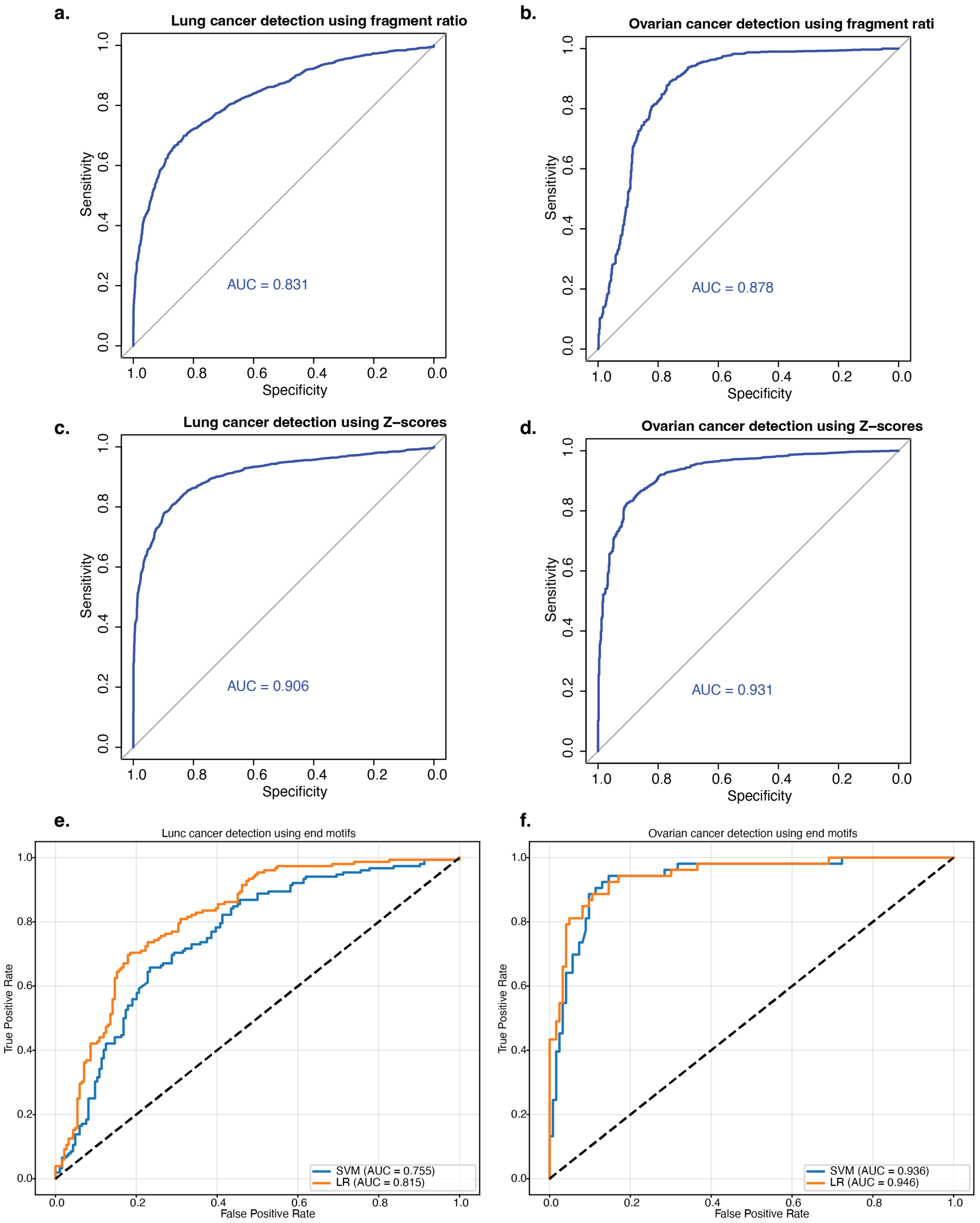

Supplemental Figure 6. Model performance metrics for cancer detection using three distinct cfDNA feature types. ROC curves for fragment ratio-based models in **a.** lung cancer and **b.** ovarian cancer. Performance of coverage Z-score-based models in **c.** lung cancer **d.** and ovarian cancer. Performance of end motif frequency-based models in **e.** lung cancer and **f.** ovarian cancer. Area under the curve (AUC) values are indicated for each model.
