# Supplementary material for: Leveraging sequences missing from the human genome to diagnose cancer
Source: Commun Med (Lond). 2025 Aug 21;5:363. doi: 10.1038/s43856-025-01067-3 (PMC12371106; doi:10.1038/s43856-025-01067-3)
Supplement: Supplementary file 2 — Description of Additional Supplementary files [file 43856_2025_1067_MOESM2_ESM.pdf]

Description of Additional Supplementary files

File name: Supplementary Data 1

Description: Supplementary Data 1: Minimal recurrency thresholds and associated number of neomers per tissue type and source data for data shown in Figure 1.

File name: Supplementary Data 2

Description: Neomers from driver mutations. Number of neomers resulting from driver mutations.

File name: Supplementary Data 3

Description: Samples meta-data and composition of tested cohorts. Performance metrics for data shown in Figure 3.

File name: Supplementary Data 4

Description: Promoter sequences cloned for luciferase assays. Genomic coordinates retrieved from hg38 genome. Reference and neomer mutation shown within parentheses.

File name: Supplementary Data 5

Description: Results of MPRA and luciferase assay.

File name: Supplementary Data 6

Description: Neomers resulting in neoantigens.
