# Supplementary material for: Leveraging sequences missing from the human genome to diagnose cancer
Source: Commun Med (Lond). 2025 Aug 21;5:363. doi: 10.1038/s43856-025-01067-3 (PMC12371106; doi:10.1038/s43856-025-01067-3)
Supplement: Supplementary file 4 — Reporting summary [file 43856_2025_1067_MOESM4_ESM.pdf]

nature portfolio

Corresponding author(s): *Double-anonymous peer review submissions: write DAPR and your manuscript number here instead of author names.*

Last updated by author(s): *YYYY-MM-DD*

Reporting Summary

Nature Portfolio wishes to improve the reproducibility of the work that we publish. This form provides structure for consistency and transparency in reporting. For further information on Nature Portfolio policies, see our Editorial Policies and the Editorial Policy Checklist.

Statistics

For all statistical analyses, confirm that the following items are present in the figure legend, table legend, main text, or Methods section.

n/a	Confirmed	
☐	☒	The exact sample size (*n*) for each experimental group/condition, given as a discrete number and unit of measurement
☐	☒	A statement on whether measurements were taken from distinct samples or whether the same sample was measured repeatedly
☐	☒	The statistical test(s) used AND whether they are one- or two-sided<br>*Only common tests should be described solely by name; describe more complex techniques in the Methods section.*
☒	☐	A description of all covariates tested
☐	☒	A description of any assumptions or corrections, such as tests of normality and adjustment for multiple comparisons
☐	☒	A full description of the statistical parameters including central tendency (e.g. means) or other basic estimates (e.g. regression coefficient) AND variation (e.g. standard deviation) or associated estimates of uncertainty (e.g. confidence intervals)
☐	☒	For null hypothesis testing, the test statistic (e.g. *F*, *t*, *r*) with confidence intervals, effect sizes, degrees of freedom and *P* value noted<br>*Give P values as exact values whenever suitable.*
☒	☐	For Bayesian analysis, information on the choice of priors and Markov chain Monte Carlo settings
☒	☐	For hierarchical and complex designs, identification of the appropriate level for tests and full reporting of outcomes
☐	☒	Estimates of effect sizes (e.g. Cohen's *d*, Pearson's *r*), indicating how they were calculated

Our web collection on statistics for biologists contains articles on many of the points above.

Software and code

Policy information about availability of computer code

Data collection	MPRAflow was used to analyze MPRA sequencing data. Somatic indel calls were performed using three pipelines from the Wellcome Sanger Institute, the DKFZ/EMBL, and the Broad Institute.
Data analysis	Custom software was used for analysis of neomers.

For manuscripts utilizing custom algorithms or software that are central to the research but not yet described in published literature, software must be made available to editors and reviewers. We strongly encourage code deposition in a community repository (e.g. GitHub). See the Nature Portfolio guidelines for submitting code & software for further information.

Data

Policy information about availability of data

All manuscripts must include a data availability statement. This statement should provide the following information, where applicable:

- Accession codes, unique identifiers, or web links for publicly available datasets
- A description of any restrictions on data availability
- For clinical datasets or third party data, please ensure that the statement adheres to our policy

WGS sequencing data was submitted to dbGAP. MPRA sequencing data was deposited in the NCBI short read archive (SRA) with accession code PRJNA917083.

Research involving human participants, their data, or biological material

Policy information about studies with human participants or human data. See also policy information about sex, gender (identity/presentation), and sexual orientation and race, ethnicity and racism.

Reporting on sex and gender	Sex determined based on self-reporting. Sex-based analysis was not relevant to our study.
Reporting on race, ethnicity, or other socially relevant groupings	N/A
Population characteristics	We obtained cfDNA samples from 201 lung cancer patients: 77 stage I (38% of cancer cases), 36 stage II (18%), 34 stage III (17%) and 53 stage IV (27%). The median age was 64+/- 7.8 years old, with patients as young as 42 years old. We also had 180 controls with a median age of 58+/- 8.1 years. Of the healthy individuals, 21% were active smokers (37.2% were noted as unknown), while 64.2% of the cancer patients were noted as active or past smoker. We also obtained cfDNA samples from 70 ovarian cancer patients with a median age of 57+/- 16.1 years: 45.7% early stage samples (24 stage I and 8 stage II), and the rest from late stage (34 stage III and 4 stage IV).
Recruitment	Participants were recruited based on disease stage and age.
Ethics oversight	UCSF human research protection program institutional review board protocol number 21-35920

Note that full information on the approval of the study protocol must also be provided in the manuscript.

Field-specific reporting

Please select the one below that is the best fit for your research. If you are not sure, read the appropriate sections before making your selection.

☒ Life sciences ☐ Behavioural & social sciences ☐ Ecological, evolutionary & environmental sciences

For a reference copy of the document with all sections, see nature.com/documents/nr-reporting-summary-flat.pdf

Life sciences study design

All studies must disclose on these points even when the disclosure is negative.

Sample size	Available samples were limited by number of samples available from biobanks and collaborators.
Data exclusions	No data were excluded from the analyses.
Replication	We used cross validation to verify reproducibility. A nested cross-validation used a 10-fold inner CV to optimize model parameters and hyperparameters and a 10-fold outer CV to test the optimized classifier on data not used for parameter and hyperparameter optimization.
Randomization	Samples were sequenced in batches of 96 and equally spread across multiple lines. Each batch contains a consistent mixture of healthy control and cancer samples.
Blinding	Sample blinding was not conducted as the knowledge of sample diagnoses was needed for training and validating the classifier.

Reporting for specific materials, systems and methods

We require information from authors about some types of materials, experimental systems and methods used in many studies. Here, indicate whether each material, system or method listed is relevant to your study. If you are not sure if a list item applies to your research, read the appropriate section before selecting a response.

Materials & experimental systems

n/a	Involved in the study	
☒	☐	Antibodies
☐	☒	Eukaryotic cell lines
☒	☐	Palaeontology and archaeology
☒	☐	Animals and other organisms
☒	☐	Clinical data
☒	☐	Dual use research of concern
☒	☐	Plants

Methods

n/a	Involved in the study	
☒	☐	ChIP-seq
☒	☐	Flow cytometry
☒	☐	MRI-based neuroimaging

Eukaryotic cell lines

Policy information about cell lines and Sex and Gender in Research

Cell line source(s)	The LNCaP cell line was purchased from ATCC
Authentication	ATCC
Mycoplasma contamination	Assayed montly in the lab.
Commonly misidentified lines (See ICLAC register)	*Name any commonly misidentified cell lines used in the study and provide a rationale for their use.*

Plants

Seed stocks	*Report on the source of all seed stocks or other plant material used. If applicable, state the seed stock centre and catalogue number. If plant specimens were collected from the field, describe the collection location, date and sampling procedures.*
Novel plant genotypes	*Describe the methods by which all novel plant genotypes were produced. This includes those generated by transgenic approaches, gene editing, chemical/radiation-based mutagenesis and hybridization. For transgenic lines, describe the transformation method, the number of independent lines analyzed and the generation upon which experiments were performed. For gene-edited lines, describe the editor used, the endogenous sequence targeted for editing, the targeting guide RNA sequence (if applicable) and how the editor was applied.*
Authentication	*Describe any authentication procedures for each seed stock used or novel genotype generated. Describe any experiments used to assess the effect of a mutation and, where applicable, how potential secondary effects (e.g. second site T-DNA insertions, mosiacism, off-target gene editing) were examined.*
